# Do expanded seven-day NHS services improve clinical outcomes? Analysis of comparative institutional performance from the *“NHS Services, Seven Days a Week”* project 2013–2016

**DOI:** 10.1186/s12913-017-2505-8

**Published:** 2017-08-10

**Authors:** Hoong-Wei Gan, Danny Jon Nian Wong, Benjamin John Floyd Dean, Alistair Scott Hall

**Affiliations:** 1 Section for Genetics & Epigenetics in Health & Disease, Genetics & Genomic Medicine Programme, University College London Institute of Child Health, 30 Guilford Street, London, WC1N 1EH UK; 2 The London Centre for Paediatric Endocrinology & Diabetes, Great Ormond Street Hospital for Children NHS Foundation Trust, London, UK; 3 National Institute of Academic Anaesthesia Health Services Research Centre, Department of Applied Health Research, University College London, London, UK; 4 Nuffield Department of Orthopaedics, Rheumatology & Musculoskeletal Sciences (NDORMS), Botnar Research Institute, Institute of Musculoskeletal Sciences, Nuffield Orthopaedic Centre, Oxford, UK; 5 Leeds MRC Medical Bioinformatics Centre, Leeds General Infirmary, Leeds, UK

**Keywords:** Hospital mortality, Length of stay, Weekend, Emergency care, Health services research

## Abstract

**Background:**

The cause of adverse weekend clinical outcomes remains unknown. In 2013, the *“NHS Services, Seven Days a Week”* project was initiated to improve access to services across the seven-day week. Three years on, we sought to analyse the impact of such changes across the English NHS.

**Methods:**

Aggregated trust-level data on crude mortality rates, Summary Hospital-Level Mortality Indicator (SHMI), mean length of stay (LOS), A&E admission and four-hour breach rates were obtained from national Hospital Episode Statistics and A&E datasets across the English NHS, excluding mental and community health trusts. Trust annual reports were analysed to determine the presence of any seven-day service reorganisation in 2013–2014. Funnel plots were generated to compare institutional performance and a difference in differences analysis was performed to determine the impact of seven-day changes on clinical outcomes between 2013 and 2014, 2014–2015 and 2015–2016. Data was summarised as mean (SD).

**Results:**

Of 159 NHS trusts, 79 (49.7%) instituted seven-day changes in 2013–2014. Crude mortality rates, A&E admission rates and mean LOS remained relatively stable between 2013 and 2016, whilst A&E four-hour breach rates nearly doubled from 5.3 to 9.7%. From 2013 to 2014 to 2014–2015 and 2015–2016, there were no significant differences in the change in crude mortality (2014–2015 *p* = 0.8, 2015–2016 *p* = 0.9), SHMI (2014–2015 *p* = 0.5, 2015–2016 *p* = 0.5), mean LOS (2014–2015 *p* = 0.5, 2015–2016 *p* = 0.4), A&E admission (2014–2015 *p* = 0.6, 2015–2016 *p* = 1.0) or four-hour breach rates (2014–2015 *p* = 0.06, 2015–2016 *p* = 0.6) between trusts that had implemented seven-day changes compared to those which had not.

**Conclusions:**

Adverse weekend clinical outcomes may not be ameliorated by large scale reorganisations aimed at improving access to health services across the week. Such changes may negatively impact care quality without additional financial investment, as demonstrated by worsening of some outcomes. Detailed prospective research is required to determine whether such reallocation of finite resources is clinically effective.

**Electronic supplementary material:**

The online version of this article (doi:10.1186/s12913-017-2505-8) contains supplementary material, which is available to authorized users.

## Background

In December 2012 National Health Service (NHS) England published *“Everyone counts: planning for patients 2013/2014”* [1], where plans for seven-day access to NHS services in the United Kingdom were outlined. This led to the establishment of an “*NHS Services, Seven Days a Week*” Forum (the “Forum”) chaired by the National Medical Director aimed at initially improving access to diagnostic, urgent and emergency services across the seven-day week.

The need for these changes were argued to be fourfold – to improve excess weekend mortality, to increase cost-efficiency, to move the NHS in line with the retail sector, and to improve the patient “customer” experience [1–3]. The Healthcare Financial Management Association (HFMA) subsequently carried out a costing analysis using a voluntary sample of eight variably sized NHS trusts at different stages of implementation (Table 1) [2]. Of these, Salford Royal, Aintree University Hospitals, Guy’s & St. Thomas’ and Chelsea & Westminster Hospital NHS Foundation Trusts had already made significant investments prior to the Forum [2, 4–8]. In 2013, both Chelsea & Westminster Hospital and Salford Royal NHS Foundation Trusts reported significant additional changes to seven-day care, whilst Guy’s & St. Thomas’ and County Durham and Darlington NHS Foundation Trusts made more minor changes [2, 5–7, 9]. Similarly, across England, other NHS trusts started reorganising services to reduce the differences in care provision across the seven-day week (full list in Additional file 1: Table S1).

**Table 1:** List of NHS Foundation Trusts included in the Healthcare Financial Management Association costing analysis, indicating services already available on a seven day basis, services invested in 2013/2014, and the potential costs of further investment in providing seven day care

NHS Trust	Already invested prior to 2013	Invested 2013/2014	Potential additional cost/year	Requiring additional investment
AUH [4]	Major trauma centre – 24/7 radiology and related trauma support services; seven day a week specialist stroke nurse service	Recruitment of two additional Critical Care Unit consultants (but not in post yet)	£5.8 million	23 additional consultants, diagnostics, therapies, pharmacy, and nursing
C&W [5]	Met most clinical standards in all specialties due to previous NHS London audits	24/7 paediatric consultant cover	£0.4 million	3 additional consultants
CRH [39, 40]	Pilot project in A&E & medicine with 6 additional consultants and support services (diagnostics, therapies, pharmacy)	None reported	£3.7 million	24 additional consultants, therapies, nursing
CD&D [9]	Little investment at assessment – not detailed	Increased Adult Mental Health Liaison services for A&E/ Medical Admissions Units 0800–2200 7 days a week	£6.5 million	15 additional consultants, diagnostics, therapies, nursing
DCH [41]	Paediatrics	None reported	£2.1 million	8 additional consultants, therapies, pharmacy, nursing
GSTT [6]	Already achieved in general medicine & vascular surgery	Improvements in specialist therapy assessment team coverage	–	–
SR [7, 8]	Increased consultant coverage in A&E (0800–2400) Emergency Assessment Unit, medical and surgical wards (0800–2000) seven days a week, increased specialist radiology 0800–2400	Opening of major trauma centre/ “emergency village” with consultant-led care (16 additional consultants) until 8 pm, therapies & pharmacy until 5 pm, seven days a week, 24/7 radiology and pathology, increasing engagement from all departments	£3.2 million	9 additional specialist consultants
WW&L [42]	Some investment at assessment – not detailed	None reported	£3.5 million	10 additional consultants, diagnostics, therapies, pharmacy, nursing

*AUH* Aintree University Hospitals NHS Foundation Trust, *C&W* Chelsea & Westminster Hospital NHS Foundation Trust, *CRH* Chesterfield Royal Hospital NHS Foundation Trust, *CD&D* County Durham & Darlington NHS Foundation Trust, *DCH* Dorset County Hospital NHS Foundation Trust, *GSTT* Guy’s & St. Thomas’ NHS Foundation Trust, *SR* Salford Royal NHS Foundation Trust, *WW&L* Wrightington, Wigan & Leigh NHS Foundation Trust

Three years on, various changes to the English NHS, including reformation of consultants’ and junior doctors’ contracts have been deemed necessary for a fully comprehensive seven-day service [10, 11]. Their basis has been primarily the excess 30-day mortality associated with weekend admissions demonstrated in two analyses of national Hospital Episode Statistics (HES) datasets from 2009 to 2010 and 2013–2014 by Freemantle et al., despite the authors stating it would be “rash and misleading” to attribute this to deficits in service provision [12, 13]. Given the three-year interval from the original Forum report, we aimed to review changes in clinical outcomes across the English NHS between 2013 and 2016, comparing NHS Trusts that had introduced seven-day changes between 2013 and 2014 and 2014–2015 to those which had not as part of what was essentially a natural intervention study. We aimed to test the null hypothesis that there were no significant differences in outcomes between these two groups during this interval.

## Methods

### Data sources

Aggregated trust-level Summary Hospital-level Mortality Indicator (SHMI; April – March 2013 to 2016) and mean length of stay (LOS; January – December 2013 to 2016) data were obtained from openly-accessible national Health & Social Care Information Centre (HSCIC) datasets (https://indicators.hscic.gov.uk/webview/, accessed 10 December 2015 and 15 November 2016), [14, 15] whilst aggregated trust-level data on Accident & Emergency (A&E) admission rates and the number of patients who had breached four-hour waiting time targets were obtained from NHS England (https://www.england.nhs.uk/statistics/statistical-work-areas/ae-waiting-times-and-activity/, accessed 10 December 2015 and 15 November 2016) [16]. The SHMI is the ratio of the observed to expected number of deaths either in-hospital or within 30 days of discharge, with the latter based on the national baseline for England, calculated from a risk-adjusted model for patient case-mix including age, gender, admission method, time of year, Charlson Comorbidity Index [17] and diagnosis [18]. The four-hour waiting time target was introduced by the Department of Health in 2004 to improve efficiency with the aim of getting 98% of patients admitted, transferred or discharged from A&E within 4 h of attending [19]. This target was relaxed to 95% in 2010.

**Fig. 1:**
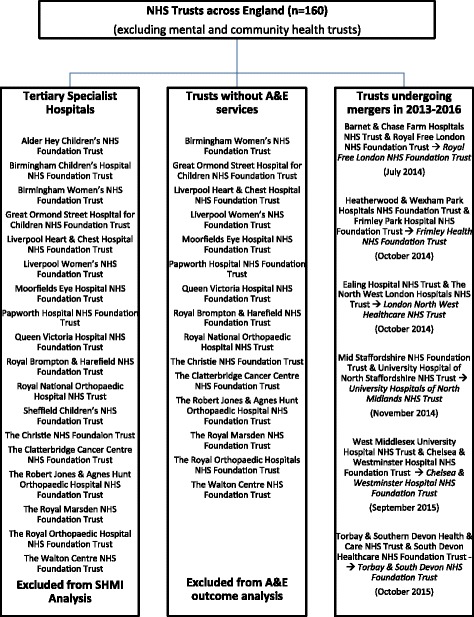
Summary of trusts excluded from various outcome metric analyses

Annual reports for 2012–2013 and 2013–2014 for each individual Trust were reviewed to determine if any seven-day service reorganisation had occurred during the period of interest. All forms of service reorganisation were considered, including weekend outpatient clinics and seven-day changes to mental and community health care, as these could have knock-on effects on admissions, inpatient bed availability, length of stay and A&E outcomes. Specialist mental health and community hospitals were excluded from all analyses (Fig. 1), whilst emergency admission and four-hour breach rate data from specialist A&Es (e.g. paediatric A&E units) was included. Due to the expected lag time from when seven-day service reorganisation was initiated in any given trust to its full implementation, we analysed outcomes over both 2014–2015 and 2015–2016 to determine their full impact.

### Statistical analysis

As the units of analysis for each outcome were individual trusts, the various outcome metrics were summarised as means (standard deviation (SD)). Funnel plots were generated to graphically compare institutional performance against other NHS Trusts across England. This method of analysis plots a clinical outcome against its precision, the latter usually proportional to the sample size [20]. Larger institutions exhibit less variation, so funnel-shaped “control” limits can be generated around the target outcome to compare like-sized institutions against each other. In these analyses, control limits corresponding to 95th (±2 SDs) and 99.8th centiles (±3 SDs) were set. Over-dispersion was accounted for by a random effects model for SHMI [18] and a generalised linear model for LOS, admission and breach rate data, both with a 10% trim (detailed methodology in Additional file 1) [20]. The difference in each of the four outcomes for each individual trust between 2013 and 2014, 2014–2015 and 2015–2016 was determined, and an unpaired t-test was used to compare the difference in differences. NHS Trusts undergoing significant restructuring through mergers with other Trusts (Fig. 1) were excluded from this analysis to provide directly comparable paired datasets. Funnel and scatter plots were generated using Microsoft Excel® for Mac 2011 version 14.6 (Microsoft Corporation, Washington). Dot and error bar plots were constructed using R version 3.2.4 (R Foundation for Statistical Computing, Vienna). Statistical analyses were performed with SPSS® Statistics version 22 (IBM Corporation, New York).

## Results

### Baseline characteristics of trusts (Fig. 1, Table 2)

Outcome data from a total of 159 trusts were analysed, with 79 (49.7%) of these instituting seven-day changes in 2013–2014. Between 2013 to 2016, 11 NHS trusts underwent mergers to form six new NHS trusts, resulting in a final number of 154 trusts by the end of 2016. Additionally, South London Healthcare NHS Trust was dissolved in October 2013, with its three constituent hospitals being taken over by King’s College Hospital NHS Foundation Trust, Lewisham and Greenwich NHS Trust, and Oxleas NHS Foundation Trust respectively (the lattermost Trust was not included in any analysis as this is a community hospital Trust with no A&E). These trusts were excluded from all pairwise difference in differences analyses. Eighteen specialist hospitals were excluded from the analysis of SHMI data due to the nature of the patients seen. Fifteen of these hospitals also did not have an A&E department and were excluded from analysis of A&E admission and four-hour breach rate data.

**Table 2:** Summary of clinical outcomes across the English NHS between 2013 to 2016

Outcome (SD)	Year					
	2013–2014	n	2014–2015	n	2015–2016	n
Crude mortality (%)	3.1 (17.4)	141	3.3 (17.8)	137	3.2 (17.6)	136
SHMI^a^	1.0 (0.1)	141	1.0 (0.1)	137	1.0 (0.1)	136
Mean LOS (days)	4.3 (1.0)	159	4.2 (0.9)	158	4.2 (1.0)	154
A&E admission rate (%)	21.8 (41.3)	145	22.1 (41.5)	140	21.8 (41.3)	138^a^
A&E four-hour breach rate (%)	5.3 (22.4)	145	7.8 (26.8)	140	9.7 (29.6)	138^a^

*SHMI* Summary Hospital Mortality Indicator, *LOS* length of stay

^a^Note that Sheffield Teaching Hospitals NHS Foundation Trust did not return A&E data for the period April 2015 – March 2016

### Summary Hospital Mortality Indicator (SHMI)

Overall annual crude mortality rates across the whole of the English NHS for the periods of April – March 2013 to 2016 were 3.1% (265,179/ 8,455,873 inpatient episodes), 3.3% (286,629/ 8,732,830 inpatient episodes), and 3.2% (282,723/ 8,825,694 inpatient episodes) respectively. Crude mortality rates over the three periods were not significantly different between Trusts that had implemented seven-day changes (*n* = 70) and those which had not (*n* = 60; mean difference: 2013–2014 to 2014–2015 + 0.2 (0.2)% vs. +0.2 (0.2)%, *p* = 0.8; 2013–2014 to 2015–2016 + 0.1 (0.3)% vs. +0.1 (0.3)%, *p* = 0.9).

**Fig. 2:**
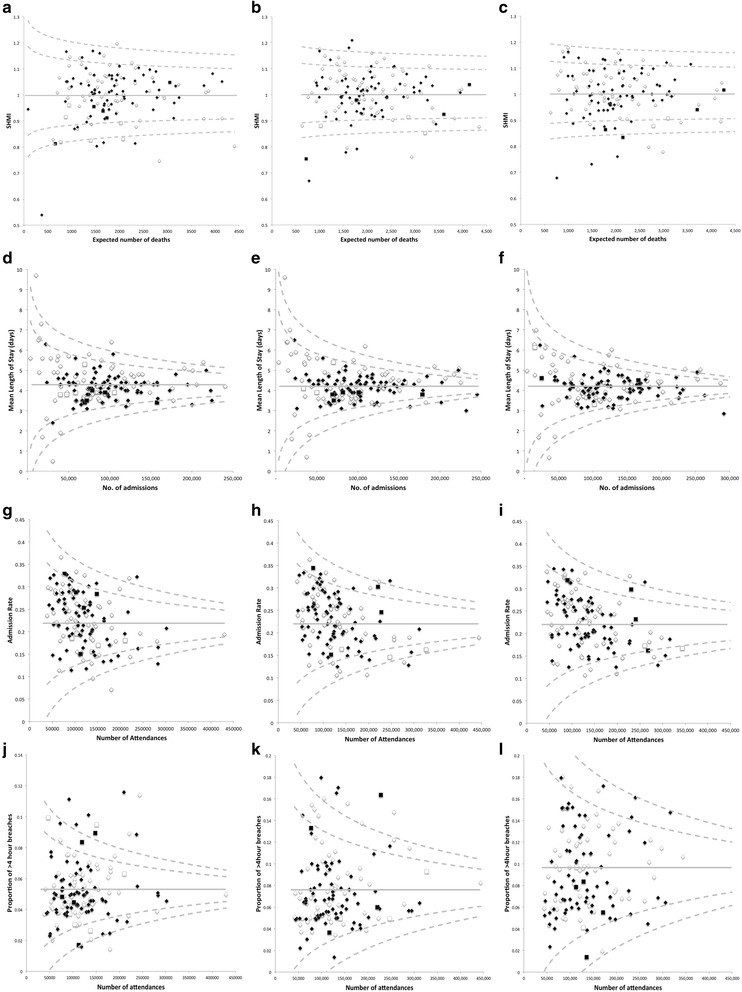
Funnel plot analyses for 2013–2014 (**a**, **d**, **g**, **j**), 2014–2015 (**b**, **e**, **h**, **k**) m and 2015–2016 (**c**, **f**, **i**, **l**) of (**a**-**c**) SHMI, (**d**-**f**) mean LOS, (**g**-**i**) A&E admission rates, and (**j**-**l**) A&E > 4 h breach rates. *Dashed lines* indicate ±2 and 3 SD control limits, whilst solid lines indicate 0 SD. Trusts implementing seven-day service changes in 2013–2014 are indicated by *solid black symbols*, those which had not are indicated by open symbols. Trusts undergoing mergers in the intervening periods are indicated by *solid black* and *open squares*

**Fig. 3:**
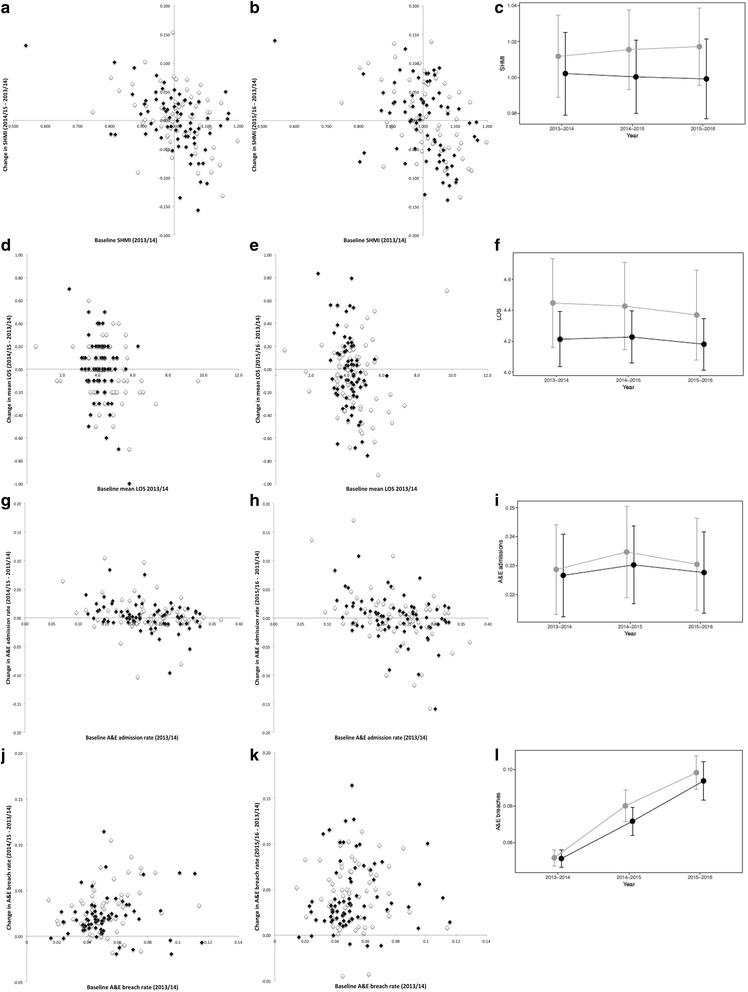
Change in (**a**-**b**) SHMI, (**d**-**e**) mean LOS, (**g**-**h**) A&E admission rates, and (**j**-**k**) A&E > 4 h breach rates between 2013 and 2014 vs. 2014–2015 and 2015–2016 plotted against baseline, with trusts instituting seven-day service changes in 2013–2014 highlighted as *solid black diamonds*, whilst those which had not indicated by open diamonds. Also shown are the changes in the mean ± 2 SE (**c**) SHMI, (**f**) mean LOS, (**i**) A&E admission rates, and (**l**) A&E > 4 h breach rates of trusts instituting seven day service changes in 2013–2014 (*solid black circles*) and those which had not (*grey circles*) over the three consecutive periods

Trusts were evenly distributed throughout the SHMI funnel plots for all periods between 2013 to 2016 regardless of whether seven-day service reorganisation had occurred or not (Fig. 2 a-c). Two trusts demonstrated an increase in SHMI beyond the +3 SD control limit for 2014–2015 despite implementing seven-day changes (Medway NHS Foundation Trust 1.17 to 1.18, North Tees & Hartlepool NHS Foundation Trust 1.16 to 1.21), whilst Blackpool Teaching Hospitals NHS Foundation Trust demonstrated an improved SHMI (1.20 to 1.16) such that it fell from above to below the +3 SD control limit between the two periods despite not undergoing any significant seven-day reorganisation. The mean differences in SHMI over the three periods were not significantly different between trusts that had implemented seven-day changes (*n* = 70) and those which had not (*n* = 60; mean difference: 2013–2014 to 2014–2015 -0.002 (0.05) vs. +0.004 (0.05), *p* = 0.5; 2013–2014 to 2015–2016 -0.003 (0.07) vs. +0.005 (0.06), *p* = 0.5; Fig. 3 a-c).

### Mean length of stay (LOS)

Average mean LOS across the English NHS was 4.3 (1.0) days for 2013–2014, 4.2 (0.9) days for 2014–2015, and 4.2 (1.0) days for 2015–2016. Once again, trusts were evenly distributed throughout the funnel plots for all three periods regardless of seven-day changes, with The Newcastle Upon Tyne NHS Hospitals NHS Foundation Trust having a mean LOS just beyond the +3 SD control limit for 2014–2015 and 2015–2016 despite such reorganisation (Fig. 2 d-f). Change in mean LOS was not significantly different over the three periods for trusts that had undergone seven-day service changes (*n* = 74) in comparison to those which had not (*n* = 74, mean difference: 2013–2014 to 2014–2015 + 0.003 (0.3) vs. -0.02 (0.2) days, *p* = 0.5; 2013–2014 to 2015–2016 -0.04 (0.3) vs. -0.08 (0.3) days, *p* = 0.4; Fig. 3 d-f).

### A&E admission rates

The proportion of patients attending English NHS A&Es who were admitted annually between April – March 2013 to 2016 was 21.8% (3,833,355/ 17,587,556 attendances), 22.1% (4,017,212/ 18,165,842 attendances) and 21.8% (4,126,579/ 18,969,653 attendances) respectively. There was no apparent change in pattern of distribution of trusts on the funnel plots over the three periods, regardless of seven-day changes, with the Heart of England NHS Foundation Trust remaining above the +3 SD control limit despite seven-day reorganisation (Fig. 2 g-i). A&E admission rates did not significantly change over the three periods for trusts that had undergone such changes (*n* = 72) in comparison to those that had not (*n* = 61; mean difference: 2013–2014 to 2014–2015 + 0.3 (2.4) vs. +0.6 (3.1)%, *p* = 0.6; 2013–2014 to 2015–2016 + 0.1 (3.9) vs. +0.1 (5.0)%, *p* = 1.0; Fig. 3 g-i).

### A&E four-hour breach rates

The proportion of English NHS A&E patients who were not admitted, transferred, or discharged in less than 4 h rose nationally year on year between April – March 2013 to 2016 (5.3% (934,770/ 17,587,556 attendances), 7.8% (1,413,830/ 18,165,842 attendances), and 9.7% (1,838,651/ 18,969,653 attendances) respectively). The distribution of trusts on the funnel plots between April 2013 to March 2014 and April 2014 to March 2015 was not apparently different between trusts which had and had not implemented seven-day changes (Fig. 2 j-k). Of the four trusts implementing seven-day changes that were above the +3 SD control limit in 2014–2015, two of these (Medway NHS Foundation Trust and Portsmouth Hospitals NHS Trust) had already been above the +3 SD control limit for 2013–2014. Hull & East Yorkshire NHS Trust experienced a large increase in the proportion of patients breaching the four-hour target (5.1% to 16.5%) such that it exceeded the +3 SD control limit for the latter period. The fourth, the University Hospitals of North Midlands NHS Trust had undergone a significant merger (previously University Hospital of North Staffordshire NHS Trust and Mid Staffordshire NHS Foundation Trust) and therefore differences in outcomes were difficult to attribute to seven-day changes. Notably, the marked widening of the funnel plots over the three periods demonstrated greater variability in trust performance with an overall worsening in breach rates, such that in 2015–2016 all trusts fell within the ±3 SD control limits (Fig. 2l). Trusts which had (*n* = 72) and had not (*n* = 61) implemented seven-day changes both experienced an annual increase in the proportion of patients breaching the four-hour target with no significant difference between the two groups (mean difference: 2013–2014 to 2014–2015 + 2.0 (2.2) vs. +2.8 (2.6)%, *p* = 0.06; 2013–2014 to 2015–2016 + 4.2 (3.7) vs. +4.5 (3.4)%, *p* = 0.6; Fig. 3 j-l).

## Discussion

The three-year lapse from the initiation of the *“NHS Services, Seven Days a Week”* Forum provided us with an opportunity to analyse outcomes from what was essentially a natural intervention trial involving all English NHS trusts, some of which had redesigned urgent, emergency and some elective services around seven-day working. The finding that for all outcomes studied, trusts instituting seven-day changes did not perform, on average, better than other institutions is unsurprising, particularly as NHS emergency services already operate 24 h a day, seven days a week. Our analysis also did not find any significant improvement in clinical outcomes over time in trusts which had actively reorganised services in 2013–2014 in comparison to those that had not. More worryingly, clinical outcomes such as A&E four-hour breach rates worsened across the English NHS despite approximately half of all trusts instituting seven-day changes.

In some organisations, despite active restructuring of services around seven-day care, there was a worsening or no change in mortality, length of stay and A&E outcomes. The reasons for this are probably multiple. Service reorganisation without sufficient additional investment or a cost-neutral budget could result in weekday care worsening at the expense of increasing weekend service provision so that overall outcomes through the seven-day week are worse or unchanged. Additionally, the association between poorer weekend clinical outcomes and service provision may potentially be non-causal, and therefore increasing weekend services may not result in improvement. Contrastingly, some trusts such as Blackpool Teaching Hospitals NHS Foundation Trust demonstrated reductions in SHMI without significant seven-day reorganisation.

The apparent association between weekend hospitalisation and increased mortality has been repeatedly demonstrated in several studies carried out in different countries [12, 13, 21–23] and healthcare settings [24]. Other outcomes such as length of stay [25] and unplanned readmission rates [26] are also higher for patients admitted over the weekend. However, the definition of what constitutes a “weekday” and the methods of case-mix adjustment in these studies have been variable, [27] and it is well-recognised that the cause of this association remains unknown. Several factors have been blamed, including reduced availability of senior staff members, diagnostic and support services [3, 13]. Other possibilities include the fact that sicker patients are more likely to present over the weekend and is incompletely adjusted for by various statistical models, or that publications surrounding the “weekend effect” are systematically biased to detect changes where there are none [28].

Recent evidence suggests that only 3% of total mortality is avoidable, [29] and several studies have now emerged suggesting that the “weekend effect” is unlikely to be improved by changes in the level of weekend staffing [30–33]. Our analysis supports this, supporting the assertion that the “weekend effect” is largely due to residual confounding and not care quality. Unlike previous studies which have not successfully determined its cause, [12, 13, 23] this analysis makes an attempt at dissecting out the effect of widespread reorganisation of clinical services around a seven-day working week on mortality, LOS and A&E quality of care, finding that these outcomes do not largely improve in trusts which have actively done so.

Despite heterogeneity in the measures taken by individual trusts to reorganise services, the changes in clinical outcomes observed between the two periods indicate these measures have also largely not improved such metrics as a whole across the English NHS. More worryingly, outcomes such as A&E four-hour breach rates were significantly worse on a background of increasing A&E attendances, suggesting increasing demands on a service where resources may have been misallocated or insufficient. We postulate therefore that large-scale changes to create a truly “seven-day NHS” such as that proposed (e.g. changes to the definition of unsociable hours working) may not lead to significant improvements, particularly in a cost-neutral setting resulting in limited weekday resources simply being moved to the weekend.

### Study limitations

We recognise that our retrospective observational analysis utilising aggregated, trust-level data from publicly available national databases of institutional performance is limited by its quality and resolution, with less capacity for adjustment of confounding to account for differences in case-mix observed at individual trusts. As such, we could only analyse crude mean LOS and A&E outcome metrics at a trust level, and any differences in these outcomes between trusts are still subject to confounding from case-mix variability. Even metrics such as the SHMI, which attempts to account for this by adjusting for comorbidities at an individual patient level using HES data, do not completely adjust for factors such as the severity of a particular comorbidity or socioeconomic status [34].

Utilisation of aggregated trust-level data also means that there is a risk of over-interpretation at an individual level, a concept known as ecological fallacy. For instance, in this study we categorised trusts as having undergone seven-day service reorganisation on the basis of any increase in seven-day provision to test the hypothesis that this would lead to improvements in clinical outcomes. A patient suffering a stroke on the weekend may have benefitted (reduced mortality risk, reduced length of stay) from some forms of seven-day service reorganisation (such as a 24/7 thrombolysis service) but not others (such as extended weekend pharmacy opening times). However, at a trust level, the limited resolution of data means that determining the clinical effectiveness of specific changes in weekend service provision at an individual level is not possible. In order to do this, further detailed interventional studies need to be undertaken, looking at the effect of a specific change in service on a specific group of patients.

Additionally, individual trusts not included in the initial HFMA costing analysis during the period of interest may not have declared the presence of seven-day service reorganisation accurately via their annual reports. It is possible that some trusts may have demonstrated improved outcomes through such reorganisation that was not formally part of the *“NHS Services, Seven Days a Week”* project, and the lack of reporting would have diluted any comparative differences in institutional performance. However, it is far more likely that given the centrally-driven push for better seven-day care, any significant changes that could potentially lead to improvements in clinical outcome would have been declared explicitly. The heterogeneity of the various measures taken by individual trusts also means that reorganisation was not uniform; however, one would expect that such a global restructuring of services should at least have resulted in measurable change in clinical outcomes when examined across the entire English NHS. We also recognise that service reorganisation takes time, and that whilst our inclusion of the 2015–2016 analysis period somewhat mitigates for the time lag in the implementation of changes in health policy, further longitudinal analyses should be performed over consecutive years to determine their full effect.

In the pairwise difference in differences analysis of clinical outcomes, we excluded 11 trusts that had undergone significant restructuring by merging with other trusts, as it would have been difficult to determine if any changes were likely to be due to seven-day service reorganisation or the restructuring itself. However, we recognise that merging of trusts may have occurred to improve access to better seven-day services, and it is possible that clinical outcomes in these trusts could have improved as a result of the merge. We have also not examined other outcomes such as patient satisfaction in this analysis, although a similar pilot scheme to extend weekend GP services reported that demand is significantly lower than expected [35].

## Conclusions

Regardless of funding method, all healthcare resources are finite and careful consideration needs to be given with regards to how they are distributed throughout the week. The NHS is predicted to be facing a £30 billion funding gap over the next 5 years, [36] and it is estimated that an additional £1.07–1.43 billion/ year is required to implement full seven-day emergency hospital services [37] with a further >£1 billion/ year for seven-day primary care [38]. A previous health economic analysis has already suggested that moving to comprehensive seven-day services does not fulfill National Institute for Health and Care Excellence (NICE) cost effectiveness criteria, [37] even if such changes are assumed to lead to improved clinical outcomes such as mortality rates. Whilst our analysis suggests that this may not be the case, its retrospective ecological design is not powered to test this hypothesis definitively. It does however suggest that more detailed prospective interventional studies such as cluster-randomised trials at an institutional or departmental level are still required to determine the aetiology of the “weekend effect”, and governmental health policy and reorganisation of health services aimed at mitigating this should await more solid evidence before being effected.

## Additional file

Additional file 1: List of all NHS Trusts in England (excluding mental and community health trusts) and details of any seven-day service reorganisation occurring between 2012 to 2014. (DOCX 67 kb)

## Abbreviations

A&E: Accident & Emergency
HES: Hospital episode statistics
HFMA: Healthcare Financial Management Association
HSCIC: Health & Social Care Information Centre
LOS: Length of stay
NHS: National Health Service
NICE: National Institute for Health & Care Excellence
SD: Standard deviation
SHMI: Summary hospital-level mortality indicator

## References

[1] NHS Commissioning Board (2012). Everyone counts: planning for patients 2013/2014.

[2] Healthcare Financial Management Association (2013). NHS services seven days a week forum: costing seven day services.

[3] England NHS (2013). NHS services seven days a week forum: summary of initial findings.

[4] Aintree University Hospital NHS Foundation Trust (2014). Annual report & accounts 2013/2014.

[5] Chelsea and Westminster Hospital NHS Foundation Trust (2014). Annual report & accounts 2013/2014.

[6] Guy’s and St. Thomas’ NHS Foundation Trust (2014). Annual report & accounts 2013–14.

[7] Salford Royal NHS Foundation Trust (2013). Annual report & accounts 1 April 2012–31 March 2013.

[8] Salford Royal NHS Foundation Trust (2014). Annual report & accounts 1 April 2013–31 March 2014.

[9] County Durham and Darlington NHS Foundation Trust (2014). Annual report & annual accounts 1 April 2013–31 March 2014.

[10] Hansard. HC Deb, vol. 600. pp. vol 600, col 151; 13 October 2015: vol 600, col 151.

[11] Hansard. HC Deb, vol 598. pp. vol 598, col 1101; 16 July 2015: vol 598, col 1101.

[12] Freemantle N, Ray D, McNulty D, Rosser D, Bennett S, Keogh BE, Pagano D (2015). Increased mortality associated with weekend hospital admission: a case for expanded seven day services?. BMJ.

[13] Freemantle N, Richardson M, Wood J, Ray D, Khosla S, Shahian D, Roche WR, Stephens I, Keogh B, Pagano D (2012). Weekend hospitalization and additional risk of death: an analysis of inpatient data. J R Soc Med.

[14] Health & Social Care Information Centre (2015). Hospital episode statistics, admitted patient care - England.

[15] Health & Social Care Information Centre (2015). Summary Hospital-level Mortality Indicator (SHMI) - Deaths associated with hospitalisation, England.

[16] England NHS (2015). A&E attendances and emergency admissions.

[17] Charlson ME, Pompei P, Ales KL, MacKenzie CR (1987). A new method of classifying prognostic comorbidity in longitudinal studies: development and validation. J Chronic Dis.

[18] Clinical Indicators Team (2015). Indicator specification: summary hospital-level mortality indicator.

[19] House of Commons Committee of Public Accounts: Department of Health: Improving Emergency Care in England, 16th Report of Session 2004-05. (Health Do ed. London: The Stationery Office Limited; 2005.

[20] Spiegelhalter DJ (2005). Funnel plots for comparing institutional performance. Stat Med.

[21] Aylin P, Yunus A, Bottle A, Majeed A, Bell D (2010). Weekend mortality for emergency admissions. A large, multicentre study. Qual Saf Health Care.

[22] Bell CM, Redelmeier DA (2001). Mortality among patients admitted to hospitals on weekends as compared with weekdays. N Engl J Med.

[23] Ruiz M, Bottle A, Aylin PP (2015). The Global Comparators project: international comparison of 30-day in-hospital mortality by day of the week. BMJ Qual Saf.

[24] Palmer WL, Bottle A, Aylin P (2015). Association between day of delivery and obstetric outcomes: observational study. BMJ.

[25] Ahmed A, Armstrong M, Robertson I, Morris AJ, Blatchford O, Stanley AJ. Upper gastrointestinal bleeding in Scotland 2000–2010: Improved outcomes but a significant weekend effect. World J Gastroenterol. 2015;(21):10890–7. 10.3748/wjg.v21.i38.10890 PMC4600590 26478680

[26] Auger KA, Davis MM. Pediatric weekend admission and increased unplanned readmission rates. J Hosp Med. 2015;10:743-5. 10.1002/jhm.2426 26381150

[27] Dean BJ (2015). The variability of a ‘weekday’ is very revealing. In response to: association between day of delivery and obstetric outcomes: observational study. BMJ.

[28] Mills JL (1993). Data torturing. N Engl J Med.

[29] Hogan H, Zipfel R, Neuburger J, Hutchings A, Darzi A, Black N (2015). Avoidability of hospital deaths and association with hospital-wide mortality ratios: retrospective case record review and regression analysis. BMJ.

[30] Aldridge C, Bion J, Boyal A, Chen YF, Clancy M, Evans T, Girling A, Lord J, Mannion R, Rees P, et al. Weekend specialist intensity and admission mortality in acute hospital trusts in England: a cross-sectional study. Lancet. 2016;388:178-86. 10.1016/S0140-6736(16)30442-1 PMC4945602 27178476

[31] Anselmi L, Meacock R, Kristensen SR, Doran T, Sutton M. Arrival by ambulance explains variation in mortality by time of admission: retrospective study of admissions to hospital following emergency department attendance in England. BMJ Qual Saf. 2017;26:613-21. 10.1136/bmjqs-2016-005680 PMC5537532 27756827

[32] Meacock R, Anselmi L, Kristensen SR, Doran T, Sutton M. Higher mortality rates amongst emergency patients admitted to hospital at weekends reflect a lower probability of admission. J Health Serv Res Policy. 2017;22:12-19. 10.1177/1355819616649630 PMC5482385 27255144

[33] Li L, Rothwell PM (2016). Biases in detection of apparent “weekend effect” on outcome with administrative coding data: population based study of stroke. BMJ.

[34] Majeed A, Dobson J (2015). Moving forwards with research on the “weekend effect”. BMJ Blogs.

[35] NHS England (2015). Prime Minister’s Challenge Fund: improving access to general practice - first evaluation report: October 2015.

[36] England NHS (2014). Five year forward view.

[37] Meacock R, Doran T, Sutton M (2015). What are the costs and benefits of providing comprehensive seven-day services for emergency hospital admissions?. Health Econ.

[38] Royal College of General Practitioners (2015). Royal College of General Practitioners seven day access to routine general practice - position paper.

[39] Chesterfield Royal Hospital NHS Foundation Trust (2014). Annual report & accounts 2013/2014.

[40] Chesterfield Royal Hospital NHS Foundation Trust (2014). Strategic plan document for 2014–19 (Public Summary).

[41] Dorset County Hospital NHS Foundation Trust (2014). Annual report & accounts 2013/14.

[42] Wrightington Wigan and Leigh NHS Foundation Trust (2014). Annual report & accounts 1 April 2013–31 March 2014.

