# Supplementary material for: Do expanded seven-day NHS services improve clinical outcomes? Analysis of comparative institutional performance from the “NHS Services, Seven Days a Week” project 2013–2016
Source: BMC Health Serv Res. 2017 Aug 10;17:552. doi: 10.1186/s12913-017-2505-8 (PMC5553994; doi:10.1186/s12913-017-2505-8)
Supplement: Additional file 1: — List of all NHS Trusts in England (excluding mental and community health trusts) and details of any seven-day service reorganisation occurring between 2012 to 2014. (DOCX 67 kb) [file 12913_2017_2505_MOESM1_ESM.docx]

**Additional file 1: Table S1**

| **NHS Trust** | **7-day changes** | **Already invested prior to 2013** | **Invested 2013/2014** | **Link to Annual Report 2013/2014** | **Comments** |
| --- | --- | --- | --- | --- | --- |
| AINTREE UNIVERSITY HOSPITAL NHS FOUNDATION TRUST | No | Major trauma centre – 24/7 radiology and related trauma support services; seven day a week specialist stroke nurse | Recruitment of two additional Critical Care Unit consultants (but not in post yet) | <http://www.aintreehospitals.nhs.uk/AboutUs/Annual%20Reports%20and%20Reviews/Annual%20Report%20and%20Accounts%202013-14.pdf> |  |
| AIREDALE NHS FOUNDATION TRUST | No | None reported | None reported | <http://www.airedale-trust.nhs.uk/Wordpress%20docs/Publications/Annual%20report/Airedale%20NHS%20Foundation%20Trust%20Annual%20Report%20and%20Accounts%202013-14.pdf> |  |
| ALDER HEY CHILDREN'S NHS FOUNDATION TRUST | No | None reported | None reported | <http://www.alderhey.nhs.uk/wp-content/uploads/Alder-Hey-Annual-Report-2013-14.pdf> |  |
| ASHFORD AND ST PETER'S HOSPITALS NHS FOUNDATION TRUST | Yes | 7-day consultant, social services and multidisciplinary team cover on Medical Assessment and Short Stay Units, weekend social work cover, elective surgical lists and outpatient clinic pilot, 6-day therapy services | 7-day radiology and surgical consultant and complex discharge team cover, patient escalation "Command Centre", weekend endoscopy lists | <http://www.ashfordstpeters.info/images/reports/201314AR.pdf> |  |
| BARKING, HAVERING AND REDBRIDGE UNIVERSITY HOSPITALS NHS TRUST | No | None reported | None reported | <http://www.bhrhospitals.nhs.uk/Annual%20Report%20and%20Accounts%202.09.2014.pdf> |  |
| BARNSLEY HOSPITAL NHS FOUNDATION TRUST | Yes | 7-day therapy services | Increased 7-day acute medical consultant cover, radiology, pharmacy, social services, transport | <http://www.barnsleyhospital.nhs.uk/uploads/2014/09/Barnsley-Hospital-NHSFT-Annual-Report-Accounts-2013_14.pdf> |  |
| BARTS HEALTH NHS TRUST | No | None reported | Executive director present on site on weekends | <http://www.bartshealth.nhs.uk/media/191185/140708%20BHT_AR_2013-14%20FINAL%20LR.pdf> |  |
| BASILDON AND THURROCK UNIVERSITY HOSPITALS NHS FOUNDATION TRUST | Yes | 24/7 paediatric A&E, increased medical consultant cover on evenings and weekends | Pilot project to increase seven-day acute & general medical and orthopaedic services with increased consultant cover | <http://www.basildonandthurrock.nhs.uk/trust-documents-and-declarations/annual-reports-and-accounts?download=724:annual-report-and-accounts-201314> |  |
| BEDFORD HOSPITAL NHS TRUST | Yes | Increased weekend consultant presence, extended diagnostics and pharmacy, "seven-day discharge arrangements" | Increased "seven-day working" and consultant cover | <https://www.bedfordhospital.nhs.uk/wp-content/upload_folder/bedford_uploads/publications/annual%20report.zip> |  |
| BIRMINGHAM CHILDREN'S HOSPITAL NHS FOUNDATION TRUST | Yes | 24/7 telephone support and weekend drop in diabetic clinic service | 7-day CAMHS Emergency Response and Assessment cover, weekend MRI service | <http://www.bch.nhs.uk/sites/bch/files/annual_report_2013-14_print_low_res_2_0.pdf> |  |
| BIRMINGHAM WOMEN'S NHS FOUNDATION TRUST | No | None reported | None reported | <http://www.bwnft.nhs.uk/wp-content/uploads/2014/10/BWNFT-Annual-Report-2013_2014-v3.pdf> |  |
| BLACKPOOL TEACHING HOSPITALS NHS FOUNDATION TRUST | No | None reported | None reported | http://www.bfwh.nhs.uk/wp-content/uploads/2015/07/Annual-Report-2013-14.pdf |  |
| BOLTON NHS FOUNDATION TRUST | No | Increased weekend consultant ward rounds, 7-day Rapid Assessment, Interface and Discharge (RAID) mental health service | None reported | <http://www.boltonft.nhs.uk/wp-content/uploads/2012/10/Annual-Report-and-Accounts-2013-14.pdf> |  |
| BRADFORD TEACHING HOSPITALS NHS FOUNDATION TRUST | Yes | Weekend stroke service | 7-day pharmacy | <https://www.bradfordhospitals.nhs.uk/uploads/uploaded_file/bradford_teaching_hospitals_nhsft_annual_report_2014-20140918131933.pdf> |  |
| BRIGHTON AND SUSSEX UNIVERSITY HOSPITALS NHS TRUST | No | None reported | None reported | https://www.bsuh.nhs.uk/EasySiteWeb/GatewayLink.aspx?alId=523562 |  |
| BUCKINGHAMSHIRE HEALTHCARE NHS TRUST | Yes | 7-day critical care outreach and transient ischaemic attack services, 24/7 stroke thrombolysis service | Increased weekend medical cover with daily patient review for weekend admissions | http://www.buckshealthcare.nhs.uk/Downloads/annualreports/p%20Annual%20report%202013-2014.pdf |  |
| BURTON HOSPITALS NHS FOUNDATION TRUST | No | 7-day occupational therapy, 24/7 thrombolysis service | None reported | <http://www.burtonhospitals.nhs.uk/FOI/Annual-Reports/2013-14/BHFT%20Annual%20Report%202013-2014.pdf> |  |
| CALDERDALE AND HUDDERSFIELD NHS FOUNDATION TRUST | Yes | N/A | 24/7 Rapid Assessment, Interface & Discharge mental health service, increased weekend patient care (not specified) | <http://www.cht.nhs.uk/fileadmin/site_setup/contentUploads/Annual_Report_and_Accounts_2013_2014.pdf> |  |
| CAMBRIDGE UNIVERSITY HOSPITALS NHS FOUNDATION TRUST | No | None reported | None reported | <http://www.cuh.org.uk/sites/default/files/CUH%20Annual%20Report%202013%202014.pdf> |  |
| CENTRAL MANCHESTER UNIVERSITY HOSPITALS NHS FOUNDATION TRUST | Yes | 7-day A&E consultant cover | 7-day stroke service and pharmacy cover | <http://www.cmft.nhs.uk/media/1049407/annual%20report%202014.pdf> |  |
| CHELSEA AND WESTMINSTER HOSPITAL NHS FOUNDATION TRUST | Yes | Chelsea & Westminster: Met most clinical standards in all specialties due to previous NHS London audits  West Middlesex: None reported | 24/7 paediatric consultant cover | <http://www.chelwest.nhs.uk/about-us/links/CW-Annual-Report-2013-14.pdf>  <http://www.chelwest.nhs.uk/about-us/links/wmuh-annual-report-2013-14.pdf> | West Middlesex University Hospital NHS Trust merged with Chelsea & Westminster Hospital NHS Foundation Trust |
| CHESTERFIELD ROYAL HOSPITAL NHS FOUNDATION TRUST | No | Pilot project in A&E & medicine with 6 additional consultants and support services (diagnostics, therapies, pharmacy) | None reported | <http://www.chesterfieldroyal.nhs.uk/news/annualreport/areport/2013_2014_annual_report?display=original> |  |
| CITY HOSPITALS SUNDERLAND NHS FOUNDATION TRUST | No | Weekend TIA clinic, 24/7 critical care outreach team | None reported | <http://chsft.nhs.uk/wp-content/uploads/2013/01/105912_CHS_Annual_Report_Inner_2013_14_low.pdf> |  |
| COLCHESTER HOSPITAL UNIVERSITY NHS FOUNDATION TRUST | Yes | Increased 7-day radiology, therapies, matrons, palliative care, Saturday outpatients | Continued expansion of 7-day palliative care, 7-day total parenteral nutrition availability for NICU | <http://www.colchesterhospital.nhs.uk/annual_reports/Annual%20Report%202013%20-%202014.pdf> |  |
| COUNTESS OF CHESTER HOSPITAL NHS FOUNDATION TRUST | No | None reported | None reported | <http://www.coch.nhs.uk/media/94230/2013_2014_ann_rep_and_accts_section_screen.pdf> |  |
| COUNTY DURHAM AND DARLINGTON NHS FOUNDATION TRUST | Yes | Little investment at assessment – not detailed | Increased Adult Mental Health Liaison services for A&E/ Medical Admissions Units 0800-2200 7 days a week | <http://www.cddft.nhs.uk/media/410781/cddft%20annual%20report%20-%20accs%202013-14%20(low%20res%20version2).pdf> |  |
| CROYDON HEALTH SERVICES NHS TRUST | No | None reported | None reported | <http://www.croydonhealthservices.nhs.uk/Downloads/Annual%20Reports/Croydon%20Health%20Services%20Annual%20Report%20and%20Accounts%202013-2014.pdf> |  |
| DARTFORD AND GRAVESHAM NHS TRUST | Yes | Increased 6 or 7-day consultant cover | Increased 7-day pharmacy, therapy and outpatient services | <http://www.dvh.nhs.uk/EasySiteWeb/GatewayLink.aspx?alId=402603> |  |
| DERBY HOSPITALS NHS FOUNDATION TRUST | Yes | None reported | 7-day single point of access Frail Elderly Assessment Pathway for GPs, Service Navigation Admission Avoidance service, consultant ward rounds, psychiatry liaison service, weekend primary care services in A&E | <http://www.derbyhospitals.nhs.uk/EasysiteWeb/getresource.axd?AssetID=172549&type=full&servicetype=Attachment> |  |
| DONCASTER AND BASSETLAW HOSPITALS NHS FOUNDATION TRUST | Yes | 7-day Assessment and Treatment Centre with consultant, pharmacy, therapy and diagnostic support services and 7-day consultant ward rounds | Continued expansion of 7-day MRI services, emergency pathways, and palliative care service | <http://www.dbh.nhs.uk/Library/Corporate_pdf_Documents/Annual%20Report%20Summary%20Financial%20Statements.pdf> |  |
| DORSET COUNTY HOSPITAL NHS FOUNDATION TRUST | No | Paediatrics | None reported | <http://www.dchft.nhs.uk/about/trust-board/Documents/annual-report-2013-14.pdf> |  |
| EAST AND NORTH HERTFORDSHIRE NHS TRUST | No | 7-day palliative care service | None reported | <http://www.enherts-tr.nhs.uk/files/2010/03/2013-14-annual-report-and-accounts-approved-and-website-ready-13-Jun-14.pdf> |  |
| EAST CHESHIRE NHS TRUST | No | None reported | None reported | <http://www.eastcheshire.nhs.uk/Downloads/ECT%20Annual%20Report%20and%20Quality%20Account%201314.pdf> |  |
| EAST KENT HOSPITALS UNIVERSITY NHS FOUNDATION TRUST | No | 7-day radiology | 6-day outpatient clinics | http://www.ekhuft.nhs.uk/EasySiteWeb/GatewayLink.aspx?alId=402882 |  |
| EAST LANCASHIRE HOSPITALS NHS TRUST | No | None reported | None reported | <http://www.elht.nhs.uk/Downloads-docs/Corporate/2014/ELHT_annual_report_final2014.pdf> |  |
| EAST SUSSEX HEALTHCARE NHS TRUST | No | None reported | None reported | <http://www.esht.nhs.uk/EasysiteWeb/getresource.axd?AssetID=506162&type=full&servicetype=Attachment> |  |
| EPSOM AND ST HELIER UNIVERSITY HOSPITALS NHS TRUST | No | None reported | None reported | <https://www.epsom-sthelier.nhs.uk/download.cfm?doc=docm93jijm4n241.pdf&ver=241> |  |
| FRIMLEY HEALTH NHS FOUNDATION TRUST | Yes | Frimley Park: Increased evening and 7-day consultant cover for most departments, and increased vascular surgery & interventional radiology services Heatherwood & Wexham Park: 7-day hip fracture theatre lists, therapies and radiology | Frimley Park: Continued expansion of 7-day consultant cover in A&E, vascular surgery, trauma & orthopaedics, cardiology and general medicine, expansion of 7-day paediatric services and cancer nurse cover Heatherwood & Wexham Park: 7-day radiology, phlebotomy, orthopaedics and cardiology, pilot 7-day speech and language therapy | <https://www.fhft.nhs.uk/media/1049/annualreport2013-14.pdfhttps://www.fhft.nhs.uk/media/1066/hwp-annual-report-accounts-2013_14.pdf> | Heatherwood & Wexham Park Hospitals NHS Trust merged with Frimley Health NHS Foundation Trust |
| GATESHEAD HEALTH NHS FOUNDATION TRUST | Yes | Opening of 24/7 early pregnancy assessment unit, 7-day mental health liaison, drugs & alcohol service | Increased 7-day medical staffing and "Lead Acute Physician" on weekends, 7-day respiratory nurse support | <http://www.qegateshead.nhs.uk/sites/default/files/users/user10/Gateshead%20Health%20NHSFT%20Annual%20Report%20and%20Accounts%20201314.pdf> |  |
| GEORGE ELIOT HOSPITAL NHS TRUST | Yes | None reported | 7-day consultant cover (increased recruitment), radiology, pharmacy and therapies, opening of new Ambulatory Medical Unit | <http://www.geh.nhs.uk/EasySiteWeb/GatewayLink.aspx?alId=4578> |  |
| GLOUCESTERSHIRE HOSPITALS NHS FOUNDATION TRUST | **No** | None reported | Virtual 7-day community multidisciplinary rapid response teams | <http://www.gloshospitals.nhs.uk/SharePoint3/Communications%20Web%20Documents/publications/Annual%20Report%202013%20-%2014%20Final%20Complete.pdf> |  |
| GREAT ORMOND STREET HOSPITAL FOR CHILDREN NHS FOUNDATION TRUST | No | None reported | 7-day Respiratory Sleep Unit* | <http://www.gosh.nhs.uk/file/8251/download?token=TRgm5A3W> |  |
| GREAT WESTERN HOSPITALS NHS FOUNDATION TRUST | Yes | Weekend discharge registrar presence | Weekend endoscopy, 6-day A&E consultant cover, pilot weekend therapy services | <http://www.gwh.nhs.uk/media/165694/final_annual_report___accounts_2013-14.pdf> |  |
| GUY'S AND ST THOMAS' NHS FOUNDATION TRUST | Yes | Already achieved in general medicine & vascular surgery | Improvements in specialist therapy assessment team coverage | <http://www.guysandstthomas.nhs.uk/resources/publications/annual-reports/2013-14-annual-report.pdf> |  |
| HAMPSHIRE HOSPITALS NHS FOUNDATION TRUST | Yes | Some 6-day radiology services | 7-day therapy services across multiple specialties, 7-day GP in A&E services, pilot 7-day consultant and pharmacy cover | <http://www.hampshirehospitals.nhs.uk/media/290445/final_annual_report_201314_-_as_sent_to_monitor_30_05_14.pdf> |  |
| HARROGATE AND DISTRICT NHS FOUNDATION TRUST | Yes | 24/7 stroke thrombolysis service | Increased 7-day endoscopy, radiology, weekend consultant physician ward rounds, electronic handover system | <http://www.hdft.nhs.uk/EasysiteWeb/getresource.axd?AssetID=9496&type=full&servicetype=Attachment> |  |
| HEART OF ENGLAND NHS FOUNDATION TRUST | Yes | Increased weekend orthogeriatric cover, 24/7 resident trauma registrar on-call | Early reconfiguration of stroke pathways to allow for 24/7 cover | <http://www.heartofengland.nhs.uk/wp-content/uploads/annual-report-_lowres.pdf> |  |
| HINCHINGBROOKE HEALTH CARE NHS TRUST | No | None reported | None reported | <http://www.hinchingbrooke.nhs.uk/wp-content/uploads/2015/02/CP0276_Hinchingbrooke-annual-report-201314_final_small.pdf> |  |
| HOMERTON UNIVERSITY HOSPITAL NHS FOUNDATION TRUST | No | 7-day breastfeeding support advisors | None reported | <http://www.homerton.nhs.uk/media/236247/Homerton-AReport-2013-14-FINAL.pdf> |  |
| HULL AND EAST YORKSHIRE HOSPITALS NHS TRUST | Yes | None reported | 7-day pharmacy, weekend eye clinics | <https://www.hey.nhs.uk/wp/wp-content/uploads/2016/03/annualReport1314.pdf> |  |
| IMPERIAL COLLEGE HEALTHCARE NHS TRUST | No | 24/7 urgent care walk-in centre | None reported | <https://www.imperial.nhs.uk/~/media/about-us/who-we-are/publications/annual-report-201314.pdf?la=en> |  |
| IPSWICH HOSPITAL NHS TRUST | No | None reported | None reported | <http://www.ipswichhospital.nhs.uk/aboutourhospital/Documents/Annual%20Reports/Annual%20Report%202013_14%20for%20web.pdf> |  |
| ISLE OF WIGHT NHS TRUST | No | None reported | None reported | <http://www.iow.nhs.uk/Downloads/Annual%20Report/Annual%20Report%20and%20Accounts%202013-14.pdf> |  |
| JAMES PAGET UNIVERSITY HOSPITALS NHS FOUNDATION TRUST | No | None reported | None reported (bid for national pilot and early adopter of NHS Seven Day Services Transformational Improvement Programme but no changes made 2013/14) | <http://www.jpaget.nhs.uk/media/226147/JPUH-Annual-Report-2013-14-web.pdf> |  |
| KETTERING GENERAL HOSPITAL NHS FOUNDATION TRUST | Yes | N/A | Extension of Ambulatory Care Unit to 7-day, weekend discharge team, pilot 7-day GP in A&E service and therapy services | <http://www.kgh.nhs.uk/EasySiteWeb/GatewayLink.aspx?alId=11602> |  |
| KING'S COLLEGE HOSPITAL NHS FOUNDATION TRUST | No | 7-day nurse-led home oxygen outpatient clinic | None reported | <https://www.kch.nhs.uk/Doc/corp%20-%20341.1%20-%20kch%20annual%20report%2013-14.pdf> |  |
| KINGSTON HOSPITAL NHS FOUNDATION TRUST | Yes | None reported | A&E & O&G consultant presence 16 hours a day, 7 days a week | <https://www.kingstonhospital.nhs.uk/media/93402/khft-annual-report-and-accounts-2013-14-parliament-.pdf> |  |
| LANCASHIRE TEACHING HOSPITALS NHS FOUNDATION TRUST | Yes | None reported | Pilot 7-day community weekend treatment rooms and minor injury units | <https://www.lancashirecare.nhs.uk/media/Publications/Annual%20Plans-Accounts-Reports/Annual%20Report%202014/Annual%20Report%20and%20Accounts%20Full.pdf> |  |
| LEEDS TEACHING HOSPITALS NHS TRUST | No | None reported | None reported | <http://www.leedsth.nhs.uk/fileadmin/Documents/About_us/Trust_Documents/Annual_Reports/Annual_Report_Summary_Account_2013.pdf> |  |
| LEWISHAM AND GREENWICH NHS TRUST | No | N/A | None reported | <http://www.lewishamandgreenwich.nhs.uk/download.cfm?doc=docm93jijm4n912.pdf&ver=1085> |  |
| LIVERPOOL HEART AND CHEST HOSPITAL NHS FOUNDATION TRUST | No | None reported | None reported | <https://www.gov.uk/government/uploads/system/uploads/attachment_data/file/450107/LIVERPOOLHEART_Annual_Report_and_Accounts_2014-15.pdf> | Note 2013-14 report missing but 2014-15 indicates no changes |
| LIVERPOOL WOMEN'S NHS FOUNDATION TRUST | No | None reported | None reported | <http://www.liverpoolwomens.nhs.uk/Library/about_us/Annual_reports/Annual_Report_2013-2014.pdf> |  |
| LONDON NORTH WEST HEALTHCARE NHS TRUST | No | Hillingdon Community Rapid Response Team (admission prevention), mental health triage units, weekend urgent advice mental health phoneline | None reported | <http://www.lnwh.nhs.uk/EasySiteWeb/GatewayLink.aspx?alId=2424http://www.lnwh.nhs.uk/EasySiteWeb/GatewayLink.aspx?alId=2824> | Previously North West London Hospitals NHS Trust, Ealing Hospital, Central Middlesex and Northwick Park Hospitals |
| LUTON AND DUNSTABLE UNIVERSITY HOSPITAL NHS FOUNDATION TRUST | Yes | Acute medical consultant cover 14 hours a day, seven days a week; seven-day CT and weekend ultrasound, social work, therapists, "navigator nurse", pharmacy; weekend outpatient clinics | Weekend consultant ward rounds on Medical Short Stay Unit, Respiratory and Cardiac Wards, completion of seven-day work in radiology (CT & MRI), pharmacy and therapies and specialties (O&G, paediatrics, emergency medicine (consultant cover 0800-2400 daily)); extension of opening hours of paediatric A&E to 24/7; continued increase in weekend outpatient clinics and operating lists | <http://www.ldh.nhs.uk/EasySiteWeb/GatewayLink.aspx?alId=8699> |  |
| MAIDSTONE AND TUNBRIDGE WELLS NHS TRUST | No | None reported | None reported | <http://www.mtw.nhs.uk/wp-content/uploads/2015/10/Annual-Report-2013-14-v5_2-including-accounts.pdf> |  |
| MEDWAY NHS FOUNDATION TRUST | Yes | None reported | Increased weekend medical cover | <http://www.medway.nhs.uk/EasySiteWeb/GatewayLink.aspx?alId=391508> |  |
| MID CHESHIRE HOSPITALS NHS FOUNDATION TRUST | Yes | None reported | Seven-day "full range of diagnostics", increased weekend consultant cover | <http://www.mcht.nhs.uk/EasySiteWeb/GatewayLink.aspx?alId=9234> |  |
| MID ESSEX HOSPITAL SERVICES NHS TRUST | No | 7-day nurse-led palliative care | None reported | <http://www.meht.nhs.uk/EasysiteWeb/getresource.axd?AssetID=10441&type=full&servicetype=Attachment> |  |
| MID YORKSHIRE HOSPITALS NHS TRUST | Yes | None reported | Extra weekend beds over winter, GP support in A&E over weekends | <https://www.midyorks.nhs.uk/download.cfm?doc=docm93jijm4n3236.pdf&ver=3546> |  |
| MILTON KEYNES HOSPITAL NHS FOUNDATION TRUST | No | None reported | None reported | <http://www.mkhospital.nhs.uk/index.php?view=download&alias=1062-2013-14-annual-report-and-accounts&category_slug=annual-reports&option=com_docman&layout=table&Itemid=646> |  |
| MOORFIELDS EYE HOSPITAL NHS FOUNDATION TRUST | No | Increased weekend Emergency Nurse Practitioner cover in A&E | None reported | <http://www.moorfields.nhs.uk/sites/default/files/Annual%20report%20and%20accounts%202013-14.pdf> |  |
| NORFOLK AND NORWICH UNIVERSITY HOSPITALS NHS FOUNDATION TRUST | Yes | None reported | Increase 7-day Immediate Assessment Unit opening hours, pilot 7-day working for therapies and social services | <http://www.nnuh.nhs.uk/publication/download/annual-report-2013-2014> |  |
| NORTH BRISTOL NHS TRUST | **No** | None reported | Weekend outpatient clinics | <https://www.nbt.nhs.uk/sites/default/files/attachments/Annual%20report%20and%20financial%20statements%202013-2014.pdf> |  |
| NORTH CUMBRIA UNIVERSITY HOSPITALS NHS TRUST | Yes | None reported | Increased agency nurse cover to support weekend working, 24/7 non-invasive ventilation service and heart centre | <http://www.ncuh.nhs.uk/about-us/our-publications/annual-reports/annual-report-2013-14.pdf> |  |
| NORTH MIDDLESEX UNIVERSITY HOSPITAL NHS TRUST | Yes | None reported | Consultant presence 16 hours a day, 7 days a week, with daily consultant ward rounds | <http://www.northmid.nhs.uk/Portals/0/North%20Middlesex%20University%20Hospital%20Annual%20report%20and%20accounts%20201314.pdf> |  |
| NORTH TEES AND HARTLEPOOL NHS FOUNDATION TRUST | Yes | None reported | Extended weekend pharmacy cover | <http://www.nth.nhs.uk/content/uploads/2014/07/annual-report-2013-2014.pdf> |  |
| NORTHAMPTON GENERAL HOSPITAL NHS TRUST | Yes | None reported | 7-day A&E consultant cover, increased nurse recruitment to cover weekends | <http://www.northamptongeneral.nhs.uk/AboutUs/Downloads/Annual-Report-2013-14-FINAL2.pdf> |  |
| NORTHERN DEVON HEALTHCARE NHS TRUST | No | None reported | None reported | <http://www.northdevonhealth.nhs.uk/wp-content/uploads/2011/10/Annual-Report-2013-14-FINAL.pdf> |  |
| NORTHERN LINCOLNSHIRE AND GOOLE NHS FOUNDATION TRUST | Yes | 7-day radiology and therapeutics including CT/MRI | 24/7 stroke and thrombolysis service, including telemedicine, expansion of 7-day radiology (ultrasound for DVT), endoscopy | <http://www.nlg.nhs.uk/content/uploads/2013/10/Annual_Report_2013-14-web.pdf> |  |
| NORTHUMBRIA HEALTHCARE NHS FOUNDATION TRUST | Yes | Increased 7-day physiotherapy services, infection control nurse and microbiology support | Increased 7-day consultant cover | <https://www.northumbria.nhs.uk/sites/all/themes/northumbria_nhs/downloads/Combined_Annual_Report_2013_-_14_Final.pdf> |  |
| NOTTINGHAM UNIVERSITY HOSPITALS NHS TRUST | No | 24/7 telemedicine supported stroke service | None reported | <http://www.nuh.nhs.uk/media/1611521/annual_report_full_version_2013-14_web.pdf> |  |
| OXFORD UNIVERSITY HOSPITALS NHS TRUST | Yes | 24/7 trauma service, weekend radiology service | 7-day radiotherapy, consultant cover in A&E, diagnostics and maternity services, supported hospital discharge service | <http://www.ouh.nhs.uk/about/publications/documents/annual-report-2014.pdf> |  |
| PAPWORTH HOSPITAL NHS FOUNDATION TRUST | Yes | Saturday occupational therapy and pharmacy services | Increased "critical care cover" on weekends | <http://www.papworthhospital.nhs.uk/docs/accounts/Papworth_Hospital_Annual_Report_Accounts_2013-14.pdf> |  |
| PENNINE ACUTE HOSPITALS NHS TRUST | Yes | None reported | 7-day stroke service | <http://www.pat.nhs.uk/about-us/Pennine%20Annual%20Report%20final.pdf> |  |
| PETERBOROUGH AND STAMFORD HOSPITALS NHS FOUNDATION TRUST | No | None reported | None reported | <https://www.peterboroughandstamford.nhs.uk/_files/4D954480A0ED3ADB9B1F1F6810E8A79F.pdf> |  |
| PLYMOUTH HOSPITALS NHS TRUST | Yes | N/A | 7-day pharmacy, forward planning for weekend nurse-led discharge | <http://www.plymouthhospitals.nhs.uk/download.cfm?doc=docm93jijm4n496.pdf&ver=624> |  |
| POOLE HOSPITAL NHS FOUNDATION TRUST | Yes | 7-day consultant ward rounds on elderly care unit with support staff (pharmacy, therapy, social services) | 7-day trauma team support from consultant in elderly care | <https://www.poole.nhs.uk/PDF/PHFT%20Annual%20report%20and%20accounts%201314%20FINAL.pdf> |  |
| PORTSMOUTH HOSPITALS NHS TRUST | Yes | None reported | 7-day ambulatory service in A&E, increased "7-day working" | <http://www.porthosp.nhs.uk/Downloads/Annual%20report%20201314.pdf> |  |
| QUEEN VICTORIA HOSPITAL NHS FOUNDATION TRUST | No | None reported | None reported | <http://www.qvh.nhs.uk/wp-content/uploads/2015/09/AR-2014.pdf> |  |
| ROYAL BERKSHIRE NHS FOUNDATION TRUST | Yes | None reported | Increased 7-day consultant and staff cover | <http://www.royalberkshire.nhs.uk/Downloads/About%20us/Annual%20Report%2013-14.pdf> |  |
| ROYAL BROMPTON & HAREFIELD NHS FOUNDATION TRUST | No | None reported | None reported | http://www.rbht.nhs.uk/EasySiteWeb/GatewayLink.aspx?alId=1244335 |  |
| ROYAL CORNWALL HOSPITALS NHS TRUST | No | None reported | None reported | <https://issuu.com/simonlloyd/docs/nhs_annual_report_online_version_fi/1> |  |
| ROYAL DEVON AND EXETER NHS FOUNDATION TRUST | Yes | None reported | 7-day paediatric assessment unit | <http://www.rdehospital.nhs.uk/docs/trust/documents/Annual%20report%20201314%20FINAL%20PUBLISHED.pdf> |  |
| ROYAL FREE LONDON NHS FOUNDATION TRUST | No | None reported | None reported | <http://s3-eu-west-1.amazonaws.com/files.royalfree.nhs.uk/Annual_report/Barnet_and_Chase_Farm_annual-report-2013-2014.pdfhttp://s3-eu-west-1.amazonaws.com/files.royalfree.nhs.uk/Annual_report/Royal_Free_London_NHS_Foundation_Trust_annual_report_2013-14.pdf> | Barnet & Chase Farm Hospitals NHS Trust merged with Royal Free London NHS Foundation Trust |
| ROYAL LIVERPOOL AND BROADGREEN UNIVERSITY HOSPITALS NHS TRUST | No | None reported | None reported | <http://www.rlbuht.nhs.uk/About%20Us/Documents/Annual%20reports/Annual%20Report%202013-14.pdf> |  |
| ROYAL NATIONAL ORTHOPAEDIC HOSPITAL NHS TRUST | No | None reported | None reported | <https://www.rnoh.nhs.uk/sites/default/files/downloads/14-133_rnoh_a4_annual_report_2014_web_release_191114.pdf> |  |
| ROYAL SURREY COUNTY HOSPITAL NHS FOUNDATION TRUST | No | None reported | None reported | <http://www.royalsurrey.nhs.uk/wp-content/uploads/2015/09/RSCH-Annual-Report-and-Accounts-2013-14-v31.pdf> |  |
| ROYAL UNITED HOSPITAL BATH NHS TRUST | No | None reported | None reported | <http://www.ruh.nhs.uk/about/annual_report/documents/Annual_Report_2013-14.pdf> |  |
| SALFORD ROYAL NHS FOUNDATION TRUST | Yes | Increased consultant coverage in A&E (0800-2400) Emergency Assessment Unit, medical and surgical wards (0800-2000) seven days a week, increased specialist radiology 0800-2400 | Opening of major trauma centre/ “emergency village” with consultant-led care (16 additional consultants) until 8pm, therapies & pharmacy until 5pm, seven days a week, 24/7 radiology and pathology, increasing engagement from all departments | <http://www.srft.nhs.uk/EasysiteWeb/getresource.axd?AssetID=34236&type=full&servicetype=Inline> |  |
| SALISBURY NHS FOUNDATION TRUST | Yes | Increased weekend therapy services and discharge coordinator cover, junior doctors developed priority bleep system for weekend working, 7-day transient ischaemic attack clinic | 7-day palliative care service | <http://www.salisbury.nhs.uk/AboutUs/TrustReportsAndReviews/Documents/AnnualReportaandAccounts20132014.pdf> |  |
| SANDWELL AND WEST BIRMINGHAM HOSPITALS NHS TRUST | Yes | Increased funding for 7-day A&E consultant cover | 7-day integrated care services team, increased physiotherapy, radiology and radiotherapy, medical consultant cover, palliative care service, trauma clinic and pharmacy, 24/7 Surgical Assessment Unit | <http://www.swbh.nhs.uk/wp-content/uploads/2014/09/NHS_Annual_Report_2014_-_FINAL.pdf> |  |
| SHEFFIELD CHILDREN'S NHS FOUNDATION TRUST | Yes | None reported | Supernumerary senior children's nurse on nights and weekends to coordinate care | <http://www.sheffieldchildrens.nhs.uk/downloads/reports/SCNHSFTAnnualReport13-14.pdf> |  |
| SHEFFIELD TEACHING HOSPITALS NHS FOUNDATION TRUST | No | 24/7 stroke consultant service via telemedicine and thrombolysis service, 24/7 primary PCI service | None reported | <http://www.sth.nhs.uk/clientfiles/File/STH%20Annual%20Report%202013-14.pdf> |  |
| SHERWOOD FOREST HOSPITALS NHS FOUNDATION TRUST | Yes | 7-day consultant cover, weekend ward rounds, emergency radiology and surgery lists | 7-day Emergency Admissions Unit pharmacy cover, increased weekend A&E cover | <http://www.sfh-tr.nhs.uk/images/aaaaaaaannn.pdf> |  |
| SHREWSBURY AND TELFORD HOSPITAL NHS TRUST | Yes | 24/7 consultant cardiology cover, increased 7-day acute medical consultant cover | 7-day radiology, pathology and therapy services | <http://www.sath.nhs.uk/Library/Documents/about/SATH-AnnualReport-1314-web.pdf> |  |
| SOUTH TEES HOSPITALS NHS FOUNDATION TRUST | Yes | Weekend outpatient clinics/ elective surgery lists | Increased weekend pharmacy cover | <http://southtees.nhs.uk/content/uploads/South-Tees-2013-2014.pdf> |  |
| SOUTH TYNESIDE NHS FOUNDATION TRUST | No | 24/7 stroke consultant service via telemedicine, and weekend high-risk transient ischaemic attack clinic | None reported | <https://www.stft.nhs.uk/repository/documents/uploaded/1411036780_annualReport13-14.pdf> |  |
| SOUTH WARWICKSHIRE NHS FOUNDATION TRUST | Yes | 7-day nurse-led admission prevention "Virtual Ward" service, A&E and AMU consultant cover, inpatient ward rounds, radiology | 7-day ambulatory emergency care clinic, community emergency response team, adult mental health cover and pharmacy | <https://www.swft.nhs.uk/media/62791/updated%20annual%20report%20-%20merged%20for%20website.pdf> |  |
| SOUTHEND UNIVERSITY HOSPITAL NHS FOUNDATION TRUST | No | None reported | None reported | <http://www.southend.nhs.uk/media/113926/41137_nhs_southend_annual_report_2014_v4.pdf> |  |
| SOUTHPORT AND ORMSKIRK HOSPITAL NHS TRUST | No | None reported | None reported | <http://www.southportandormskirk.nhs.uk/downloads/Trust-Finance/Annual-Report-13-14.pdf> |  |
| ST GEORGE'S HEALTHCARE NHS TRUST | Yes | 7-day nurse-led discharge service and paediatric consultant cover | 24/7 primary PCI service | <https://www.stgeorges.nhs.uk/wp-content/uploads/2014/10/Annual-report-sr.pdf> |  |
| ST HELENS AND KNOWSLEY HOSPITALS NHS TRUST | Yes | Increased consultant coverage especially AMU (2 new consultants), 24/7 stroke thrombolysis & plastic surgery trauma service, weekend radiology & gynaecology enhanced scanning service | 6 new consultants to improve consultant coverage with daily senior review in some specialties, weekend diabetes and other specialty clinics, rapid response Hospital at Home COPD service, opening of 7-day trauma rehabilitation unit | <http://www.sthk.nhs.uk/about/Documents/ANNUAL%20REPORT%202013%202014.pdf> |  |
| STOCKPORT NHS FOUNDATION TRUST | Yes | Extended weekend palliative care service and antibiotic pharmacist cover | Changes to medical rotas to faciliatate 7-day working, Macmillan nurse support | <https://www.stockport.nhs.uk/webdocs/NewsDocs/Stockport%20NHS%20FT%20Annual%20Report%20and%20Accounts%202013-14-Final%20for%20Parliament.pdf> |  |
| SURREY AND SUSSEX HEALTHCARE NHS TRUST | Yes | 7-day Transient Ischaemic Attack service, weekend elective surgical lists | Increased capacity in A&E with 7-day consultant cover and therapy services, increased weekend consultant and junior doctors cover in multiple medical specialties with 7-day consultant ward rounds | <http://www.surreyandsussex.nhs.uk/wp-content/uploads/2013/02/SASH-annual-report-2014-v3b-PRESS.pdf> |  |
| TAMESIDE HOSPITAL NHS FOUNDATION TRUST | Yes | 7-day radiology | 7-day pharmacy, increased weekend medical staff cover | <http://www.tamesidehospital.nhs.uk/Documents/Tamesideannualreport201314.pdf> |  |
| TAUNTON AND SOMERSET NHS FOUNDATION TRUST | No | None reported | None reported | <http://www.musgroveparkhospital.nhs.uk/media/274665/TSFT-Annual-Report-QA-plus-Account-2013-14.pdf> |  |
| THE CHRISTIE NHS FOUNDATION TRUST | No | None reported | None reported | <http://www.christie.nhs.uk/media/1607/the_christie_annual_report_and_accounts_2013-14.pdf> |  |
| THE CLATTERBRIDGE CANCER CENTRE NHS FOUNDATION TRUST | No | 24/7 chemotherapy triage service, Acute Assessment Unit and palliative care nursing | None reported | <http://www.clatterbridgecc.nhs.uk/application/files/7914/3506/9639/AnnualReportAccounts1314.pdf> |  |
| THE DUDLEY GROUP NHS FOUNDATION TRUST | No | 7-day outpatient IV antibiotic clinic, weekend daycase elective surgery | None reported | <http://dudleygroup.nhs.uk/wp-content/uploads/2014/02/The-Dudley-Group-NHS-Foundation-Trust-Annual-report-Accounts-and-Quality-Report-2013-14.pdf> |  |
| THE HILLINGDON HOSPITALS NHS FOUNDATION TRUST | Yes | Twice-daily consultant ward rounds on EAU, increase in therapy provision on the weekends | Extended cover for acute medicine, paediatrics, O&G, A&E over 7 days, enhanced therapy & diagnostic services | <http://www.thh.nhs.uk/documents/_Publications/AnnualReports/AnnualReport2014.pdf> |  |
| THE NEWCASTLE UPON TYNE HOSPITALS NHS FOUNDATION TRUST | Yes | None reported | 7-day intravenous antibiotic at home service and pharmacy | <http://www.newcastle-hospitals.org.uk/downloads/Performance%20information/2013-14_NuTH_Annual_Report_Part1.pdf> |  |
| THE PRINCESS ALEXANDRA HOSPITAL NHS TRUST | No | None reported | None reported | <http://www.pah.nhs.uk/files/file/Annual%20Report%20final.pdf> |  |
| THE QUEEN ELIZABETH HOSPITAL, KING'S LYNN, NHS FOUNDATION TRUST | Yes | None reported | Extended Rapid Assessment Team services on weekend for early discharge | <http://www.qehkl.nhs.uk/Documents/AnnualReport1314.doc> |  |
| THE ROBERT JONES AND AGNES HUNT ORTHOPAEDIC HOSPITAL NHS FOUNDATION TRUST | No | None reported | None reported | <http://www.rjah.nhs.uk/RJAHNHS/files/ed/edd63457-4ddc-4282-be35-168862505c46.pdf> |  |
| THE ROTHERHAM NHS FOUNDATION TRUST | No | None reported | None reported | <http://www.therotherhamft.nhs.uk/Corporate_Governance_Information/Annual_Report_2013_-_2014/> |  |
| THE ROYAL BOURNEMOUTH AND CHRISTCHURCH HOSPITALS NHS FOUNDATION TRUST | Yes | 24/7 percutaneous coronary intervention, 7-day discharge coordination team, hospital at night nurse coordinator, consultant physician cover | 7-day radiology, elderly care senior nurse support, beginning of move to 7-day speech and language therapy, consultant cover in A&E and elderly care | <http://www.rbch.nhs.uk/assets/templates/rbch/documents/about_the_trust/our_publications/annual_report/annualreport_2014.pdf> |  |
| THE ROYAL MARSDEN NHS FOUNDATION TRUST | Yes | N/A | 24/7 interventional radiology service | <https://www.royalmarsden.nhs.uk/sites/default/files/files_trust/annual-report-accounts-1314.pdf> |  |
| THE ROYAL ORTHOPAEDIC HOSPITAL NHS FOUNDATION TRUST | No | None reported | None reported | <http://www.roh.nhs.uk/about-us/publications/annual-report/154-annual-report-and-accounts-2013-14/file> |  |
| THE ROYAL WOLVERHAMPTON NHS TRUST | Yes | None reported | Opening of 24/7 clinical decisions unit | <http://www.royalwolverhamptonhospitals.nhs.uk/pdf/Annual%20report%20final%2030%20June%20from%20website.pdf> |  |
| THE WALTON CENTRE NHS FOUNDATION TRUST | No | None reported | None reported | <https://www.thewaltoncentre.nhs.uk/uploadedfiles/Annual%20Report%20and%20Accounts%202013-14.pdf> |  |
| THE WHITTINGTON HOSPITAL NHS TRUST | Yes | 7-day medical consultant cover, ambulatory nurse-led clinics at weekends | 7-day paediatric consultant cover, rapid response nurse-led home service to avoid admissions, hospital-at-home service, respiratory community team, integrated mental health liaison and pharmacy, opening of 7-day ambulatory care centre and increased weekend urgent care staffing | <http://www.whittington.nhs.uk/document.ashx?id=4732> |  |
| TORBAY AND SOUTH DEVON NHS FOUNDATION TRUST | Yes | Sunday consultant ward rounds, increased weekend junior doctors | Ward clerk support on Sunday ward round | <http://www.torbayandsouthdevon.nhs.uk/uploads/annual-report-and-accounts-2013-14.pdf> | Torbay & Southern Devon Health & Care NHS Trust (primary care trust) merged with South Devon Healthcare NHS Foundation Trust |
| UNITED LINCOLNSHIRE HOSPITALS NHS TRUST | No | None reported | None reported | <https://www.ulh.nhs.uk/content/uploads/2015/06/Annual_Report_2013-2014.pdf> |  |
| UNIVERSITY COLLEGE LONDON HOSPITALS NHS FOUNDATION TRUST | Yes | Increased A&E weekend medical & nursing cover | Increased weekend senior medical cover on ward rounds, 7-day specialist nurse discharge support | <http://www.uclh.nhs.uk/aboutus/wwd/Annual%20reviews%20plans%20and%20reports%20archive/Annual%20report%20and%20accounts%202013-14.pdf> |  |
| UNIVERSITY HOSPITAL OF SOUTH MANCHESTER NHS FOUNDATION TRUST | Yes | 7-day consultant cover in some departments | 7-day acute physician cover, increased 7-day theatre availability for hip fractures | <https://www.uhsm.nhs.uk/content/uploads/2015/08/4.1.2-UHSM-Annual-Report-and-Accounts-2103-2014-.pdf> |  |
| UNIVERSITY HOSPITAL SOUTHAMPTON NHS FOUNDATION TRUST | No | Launch of major trauma centre, 7-day consultant surgeon (including urology) ward rounds and on-call cover | None reported | <http://www.uhs.nhs.uk/Media/SUHTInternet/AboutUs/AnnualReportsStrategiesandPlans/20132014/Annual-report-2013-14.pdf> |  |
| UNIVERSITY HOSPITALS BIRMINGHAM NHS FOUNDATION TRUST | No | None reported | None reported | <http://www.uhb.nhs.uk/Downloads/pdf/AnnualReport13-14.pdf> |  |
| UNIVERSITY HOSPITALS BRISTOL NHS FOUNDATION TRUST | No | None reported | None reported | <http://www.uhb.nhs.uk/Downloads/pdf/AnnualReport13-14.pdf> |  |
| UNIVERSITY HOSPITALS COVENTRY AND WARWICKSHIRE NHS TRUST | No | None reported | None reported | <http://www.uhcw.nhs.uk/clientfiles/File/UHCW-2013-14-ANNUAL-REPORT-FINAL.pdf> |  |
| UNIVERSITY HOSPITALS OF LEICESTER NHS TRUST | Yes | None reported | 7-day transient ischaemic attack clinic and alcohol liaison team | <http://www.leicestershospitals.nhs.uk/EasySiteWeb/GatewayLink.aspx?alId=28421> |  |
| UNIVERSITY HOSPITALS OF MORECAMBE BAY NHS FOUNDATION TRUST | Yes | None reported | 7-day pharmacy | <https://www.uhmb.nhs.uk/files/2614/0855/3061/Annual%20Report%202013-14%20Final.pdf> |  |
| UNIVERSITY HOSPITALS OF NORTH MIDLANDS NHS TRUST | Yes | None reported | North Staffordshire: 7-day consultant O&G care (increased number of consultant obstetricians) Mid Staffordshire: None reported | <http://www.uhnm.nhs.uk/aboutus/Documents/AnnualReportsAndAccounts/UHNS%20Annual%20Report%20and%20Accounts%202013%202014.pdfhttp://www.uhnm.nhs.uk/aboutus/MSFT-PubsAndReports/MSFTPublications%20And%20Reports/HomePage-Main%20Page/Annual%20Reports%20And%20Accounts/Annual-Report-Accounts-2013-14-(inc-Quality-Accounts)-(-FINAL-).pdf> | Mid Staffordshire NHS Trust merged with University Hospitals of North Midlands NHS Trust |
| WALSALL HEALTHCARE NHS TRUST | No | None reported | None reported | <https://www.walsallhealthcare.nhs.uk/Data/Sites/1/documents/annual-report/quality-account-2013-14-(1).pdf> |  |
| WARRINGTON AND HALTON HOSPITALS NHS FOUNDATION TRUST | No | None reported | None reported | <http://www.whh.nhs.uk/_store/documents/warringtonandhaltonhospitalsnhsftannualreportandaccounts2013-2014.pdf> |  |
| WEST HERTFORDSHIRE HOSPITALS NHS TRUST | No | 7-day consultant led Rapid Assessment, Interface and Discharge mental health service and Ambulatory Care Service | None reported | <http://www.westhertshospitals.nhs.uk/annualreport/1314/58452_Annual-Report_13-14_web.pdf> |  |
| WEST SUFFOLK NHS FOUNDATION TRUST | Yes | 24/7 stroke thrombolysis service, increased 7-day consultant cover | Increased Early Intervention Team discharge planning for elderly care on the weekends increased diagnostics, implementing consultant review within 24 hours of admission, | <http://www.wsh.nhs.uk/AboutUs/TrustPublications/docs/AnnualReports/Annual-Report-and-Accounts-2013-14.pdf> |  |
| WESTERN SUSSEX HOSPITALS NHS FOUNDATION TRUST | Yes | None reported | Increased 7-day clinical and nursing cover in some specialties | <http://www.westernsussexhospitals.nhs.uk/wp-content/uploads/2014/08/WSHFT-Annual-Report-and-Accounts-2013-14.pdf> |  |
| WESTON AREA HEALTH NHS TRUST | No | None reported | None reported | <http://www.waht.nhs.uk/FOIDocuments/AnnualReportQualityAccount201314/Annual%20Report%2020132014.docx> |  |
| WIRRAL UNIVERSITY TEACHING HOSPITAL NHS FOUNDATION TRUST | Yes | Introduction of 7-day working for consultants, including radiology and 7-day medical consultant ward rounds; 7-day surgical allied health professional service; elective endoscopy lists on weekends, new 24/7 EAU | Improved 7-day working of consultant physicians, senior medical review available across week | <http://www.wuth.nhs.uk/media/684276/wuth-annual-report-2013-14.pdf> |  |
| WORCESTERSHIRE ACUTE HOSPITALS NHS TRUST | Yes | 7-day radiology and 24/7 primary PCI service | 7-day pharmacy and palliative care service, trial of weekend hip fracture and ENT operating lists | <http://www.worcsacute.nhs.uk/EasysiteWeb/getresource.axd?AssetID=62108&type=full&servicetype=Attachment> |  |
| WRIGHTINGTON, WIGAN AND LEIGH NHS FOUNDATION TRUST | No | Some investment at assessment – not detailed | None reported | <http://www.wwl.nhs.uk/Library/Trust_Board/Annual_Report/WWL_Annual_Report_2013-2014.pdf> |  |
| WYE VALLEY NHS TRUST | Yes | None reported | 7-day radiology and Clinical Assessment Unit | <http://www.wyevalley.nhs.uk/media/184391/WVT-Annual-Report-2013-14-FINAL-web-ready-July14.pdf> |  |
| YEOVIL DISTRICT HOSPITAL NHS FOUNDATION TRUST | Yes | None reported | 7-day discharge teams, pilot 7-day therapeutic pilot (e.g. TIA service) | <http://www.yeovilhospital.co.uk/wp-content/uploads/2015/07/Annual-Report-Annual-Accounts-and-Quality-Report-2013-14.pdf> |  |
| YORK TEACHING HOSPITAL NHS FOUNDATION TRUST | No | None reported | None reported | <http://www.yorkhospitals.nhs.uk/document.php?o=1008> |  |

Table 1: List of all NHS Trusts in England (excluding mental and community health trusts) and details of any seven-day service reorganisation occurring between 2012 to 2014. A&E; accident & emergency, AMU; acute medical unit, CAMHS; Child & Adolescent Mental Health Service, DVT; deep vein thrombosis, EAU; emergency admissions unit, NICU; neonatal intensive care unit, O&G; obstetrics & gynaecology, TIA; transient ischaemic attack. *Not felt to contribute towards clinical outcomes measured in this study.

**Funnel plot control limit calculations^1^**

*Mean length of stay (LOS)*

The standard error (SE) of the control limits for a given mean LOS, *Y*, about a target mean LOS (i.e. the average of all mean LOS values, θ_0_ with a standard deviation of σ) for a trust with *ρ* number of inpatient episodes was calculated as:

SE = σ/ρ^1/2^

Unadjusted control limits *y_p_* at a significance value p for the funnel plot analyses were then calculated for a given mean LOS, *Y* as:

y_p_ = θ_0_ ± z_p_(SE)

where z_0.001_ = -3, z_0.025_ = -1.96, z_0.975_ = 1.96 and z_0.999_ = 3.

However such a model involving a large sample size will almost inevitably result in over-dispersion.

In order to adjust for this we first determined the Z-scores for the mean LOS at each trust as follows:

Z = (Y - θ_0_)/σ

We then applied a 10% trim by removing trusts with Z-scores that were <-1.28 and >1.28. With this small number of trusts, we then calculated the standardised Pearson residual, z_i_ as:

z_i_ = (Y- θ_0_)/SE

And the correction factor Φ based on the reduced number of trusts, n is calculated as:

Φ = Σz_i_^2^/n

The adjusted control limits can thus be calculated as:

y_p_ = θ_0_ ± z_p_Φ^1/2^(SE)

*A&E admission & four-hour breach rates*

The method of generating adjusted control limits for A&E admission and breach rates was identical to that of mean LOS, apart from the calculation of the SE, which in this case is the standard error for a proportion:

SE = ((Y(1-Y)/ρ)^1/2^

where in this case Y is the admission or breach rate and ρ is the number of attendances at a given trust.

**References**

1. Spiegelhalter DJ. Funnel plots for comparing institutional performance. *Statistics in medicine*. 2005; 24: 1185-202.
